# Supplementary material for: Embryonic signals mediate extracellular vesicle biogenesis and trafficking at the embryo–maternal interface
Source: Cell Commun Signal. 2023 Aug 18;21:210. doi: 10.1186/s12964-023-01221-1 (PMC10436626; doi:10.1186/s12964-023-01221-1)
Supplement: Supplementary file 2 — Additional file 1: Supplementary Figure 1. Control staining performed without primary antibodies. Supplementary Figure 2. mRNA expression in luminal epithelial cells. Supplementary Figure 3. Ultrastructural changes in luminal/glandular epithelium and trophectoderm on day 20 of pregnancy. Supplementary Figure 4. Overview of micrographs showing heterogeneous population of extracellular vesicles found in endometrial compartments on Day 12 and 16 of pregnancy. Supplementary Figure 5. CD63 appearance in the endocytic membrane transport pathway. Supplementary Figure 6. miRNA effect on target gene and protein abundance. Supplementary Table 1. List of primary and secondary antibodies used in immunofluorescent staining of endometrial/trophoblast tissue and luminal epithelial cells. Supplementary Table 2. List of mimics used in miRNA and hormone treatment experiment. Supplementary Table 3. List of genes and TaqMan gene expression assays used in real-time PCR. [file 12964_2023_1221_MOESM1_ESM.pdf]

Embryonic signals mediate extracellular vesicle biogenesis and trafficking at the embryo–maternal interface

Maria M. Guzewska[1], Kamil Myszczynski[2], Yael Heifetz[3], Monika M. Kaczmarek[1,2*]

[1]Department of Hormonal Action Mechanisms; [2]Molecular Biology Laboratory, Institute of Animal Reproduction and Food Research, Polish Academy of Sciences, Olsztyn, Poland; [3]The Department of Entomology, The Hebrew University of Jerusalem, Jerusalem, Israel

Supporting Information

Figures

Supplementary Figure 1. Control staining performed without primary antibodies.

Supplementary Figure 2. mRNA expression in luminal epithelial cells.

Supplementary Figure 3. Ultrastructural changes in luminal/glandular epithelium and trophectoderm on day 20 of pregnancy.

Supplementary Figure 4. Overview of micrographs showing heterogeneous population of extracellular vesicles found in endometrial compartments on Day 12 and 16 of pregnancy.

Supplementary Figure 5. CD63 appearance in the endocytic membrane transport pathway.

Supplementary Figure 6. miRNA effect on target gene and protein abundance.

Tables

Supplementary Table 1. List of primary and secondary antibodies used in immunofluorescent staining of endometrial/trophoblast tissue and luminal epithelial cells.

Supplementary Table 2. List of mimics used in miRNA and hormone treatment experiment.

Supplementary Table 3. List of genes and TaqMan gene expression assays used in real-time PCR.

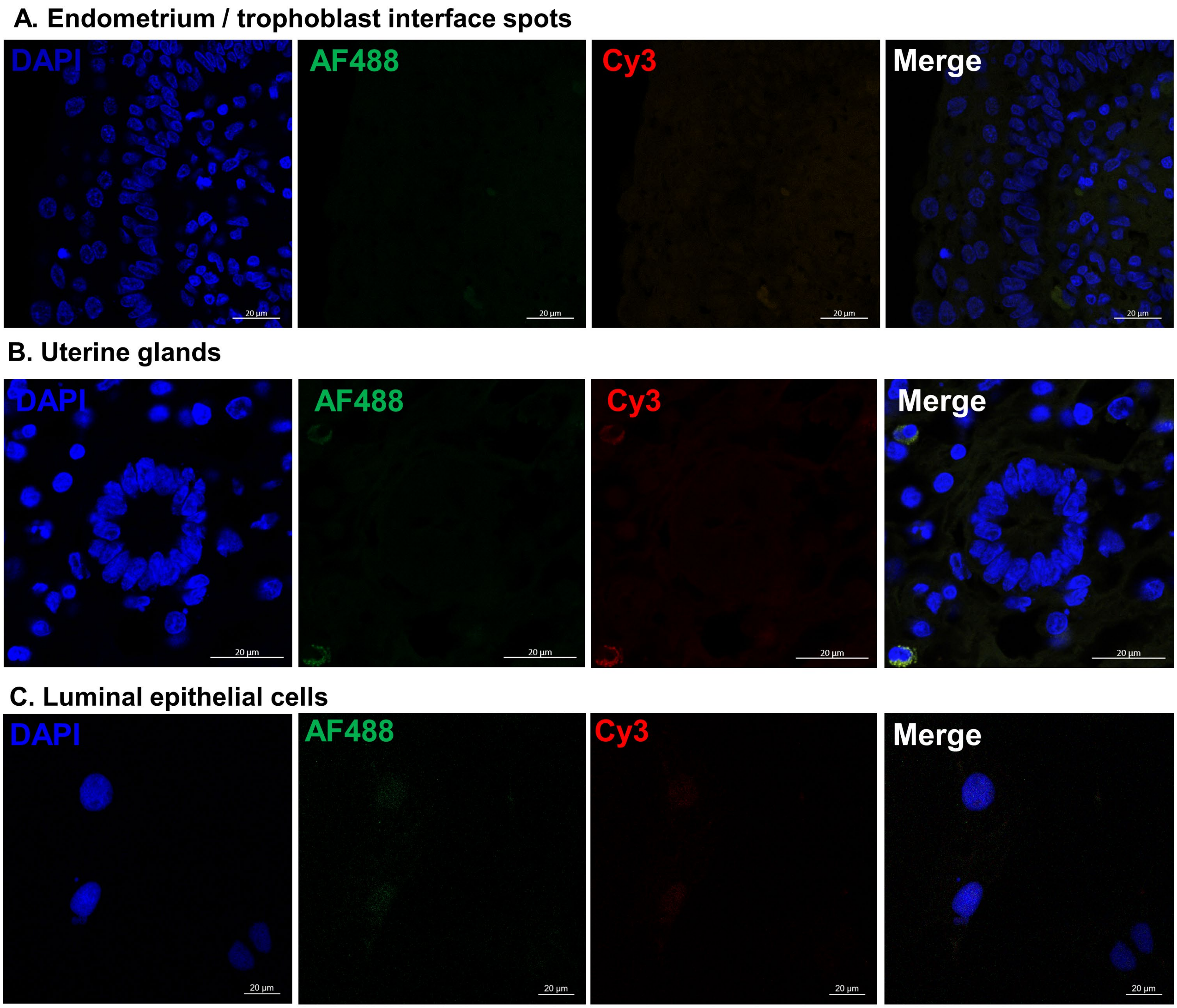

Supplementary Figure 1. Control staining performed without primary antibodies. (A) Negative control for untouched trophoblast-luminal epithelial interfaces. (B) Negative control for uterine glands. (C) Negative control for luminal epithelial cells. Merged pictures with red (Cy3-conjugated antibodies), green (Alexa Fluor 48-conjugated antibodies) and blue (DAPI) staining.

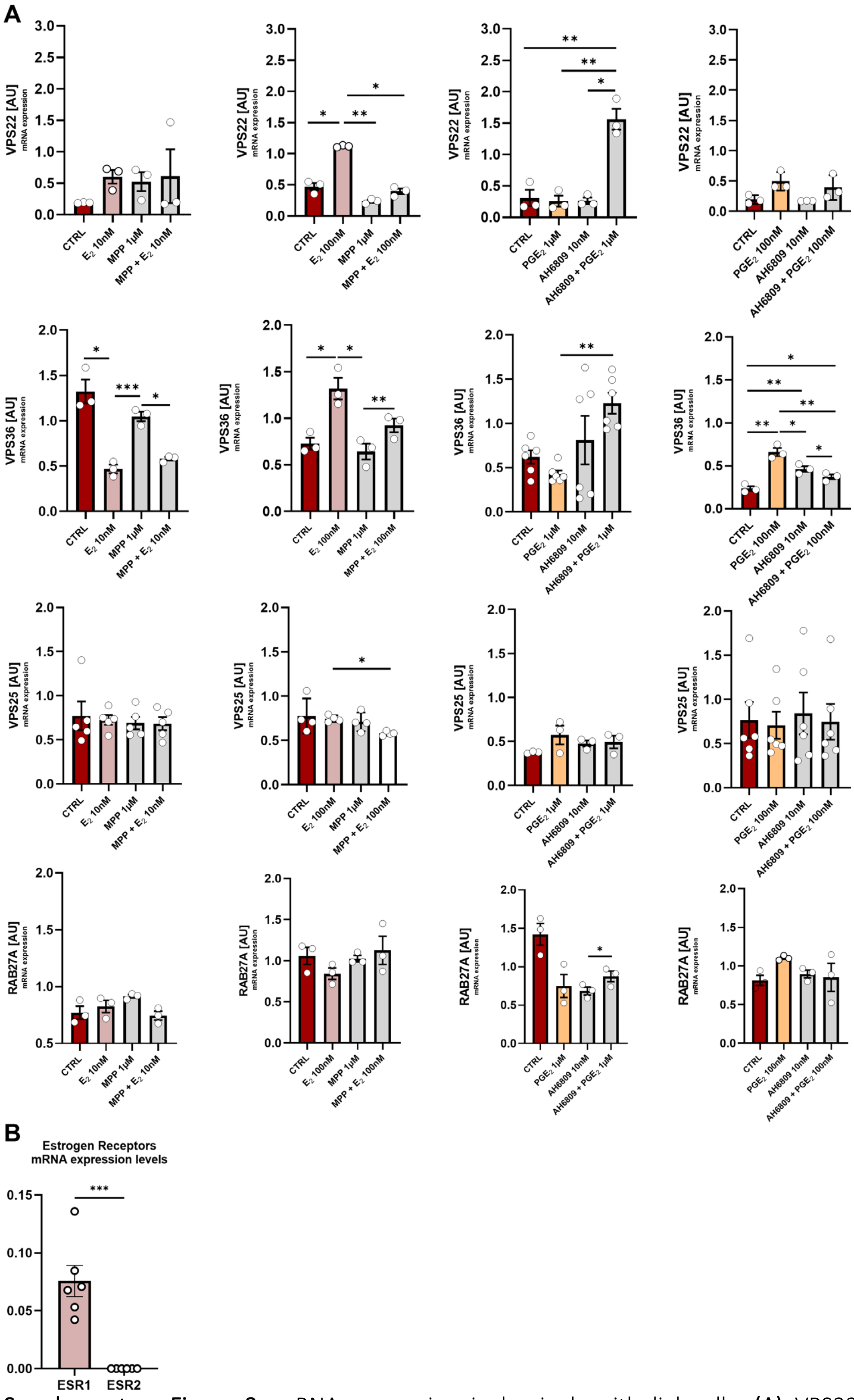

Supplementary Figure 2. mRNA expression in luminal epithelial cells. **(A)** VPS22, VPS25, VPS36, and Rab27A gene expression in response to treatment with E2 or PGE2 of primary luminal epithelial cells. Different doses of E2 (10 nM or 100 nM, pink boxplots) and PGE2 (1 µM or 100 nM, yellow boxplots) were used. Cells were pretreated with specific inhibitors – MPP for ESR1 (1h, 1 µM) or AH6809 for PGE2 (10 minutes, 10 µM). Data were analyzed using one-way ANOVA with Tukey's multiple comparison test (vs. control; * $p < 0.05$, ** $p < 0.01$, *** $p < 0.001$) and are shown as means ± SEM (n = 3 - 6). **(B)** ESR1 and ESR2 gene expression in response to treatment with E2 (100 nM). Data were analyzed using unpaired T- test (*** $p < 0.001$) and are shown as means ± SEM (n = 6). mRNA expression was normalized to HPRT1 (AU), identified as the best reference gene by NormFinder algorithm.

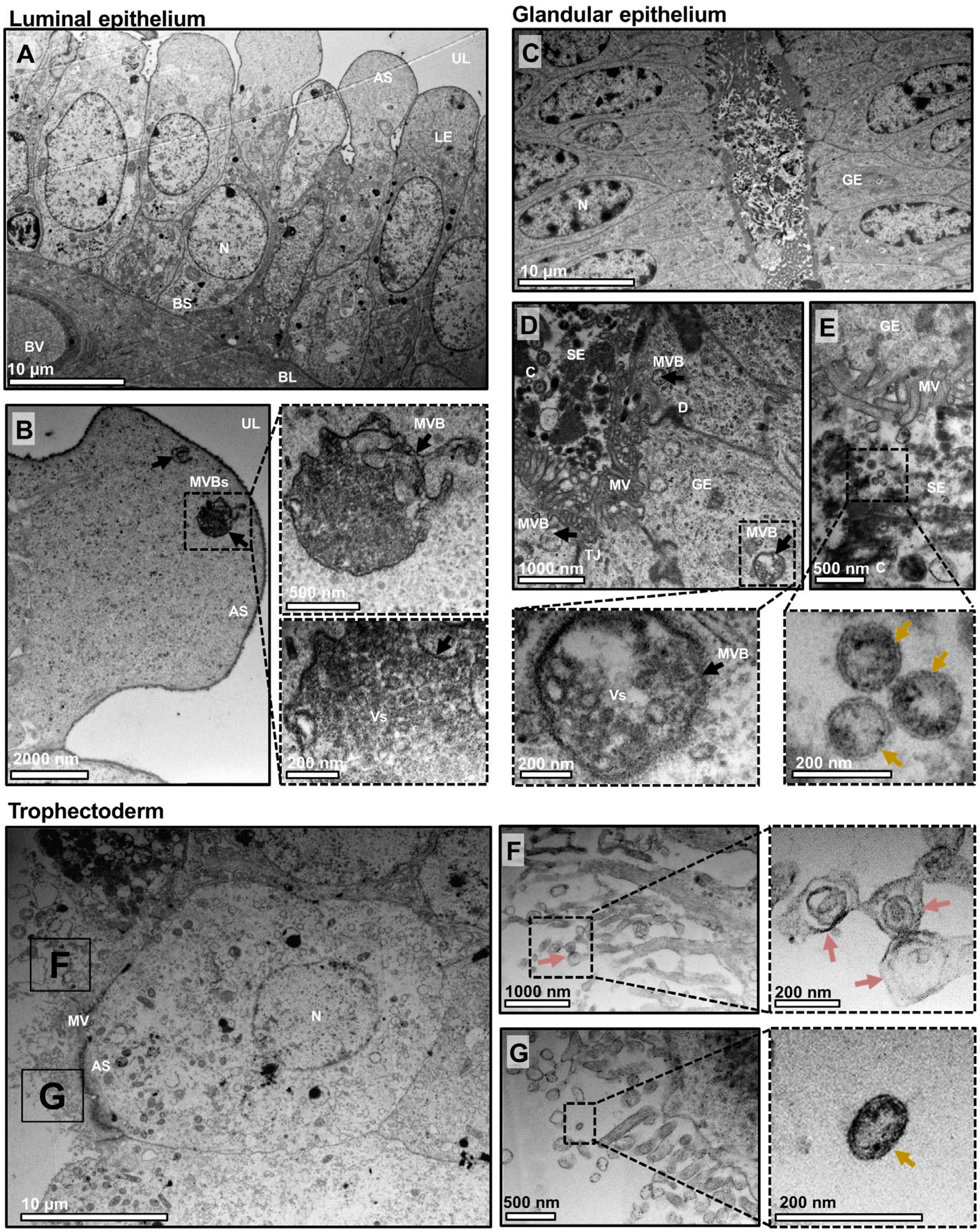

Supplementary Figure 3. Ultrastructural changes in luminal/glandular epithelium and trophectoderm on day 20 of pregnancy. **(A)** Cross section of luminal epithelium showing tall, columnar epithelial cells with large nucleus and any organelles at the apical site of the cell. **(B)** Large MVB about to fuse with plasma membrane were noticed at the apical site. MVBs contained heterogeneous population of double-membrane vesicles ready to be released into the extracellular space (higher magnification). **(C)** Cross section of wide open uterine gland. Large population of glandular cells with cilia, microvilli were observed. Glands were filled with secretome, containing heterogeneous population of EVs (yellow arrows), cached between the microvilli and cilia. The apical site of the glandular epithelial cells showed MVBs (black arrow) filled with double-membrane vesicles located under the plasma membrane (higher magnification). Lower panel: Cross sections of the trophectoderm. **(F)** Apical surface is covered with numerous microvilli. Apocrine type of secretion (pink arrows) was also observed, as the cytoplasmic fragments containing the numerous secretory vesicles (higher magnification). (G). Secreted, free double-membrane vesicles were found between the microvilli. AS – apical site, BL – basal lamina, BS – basal site, BV – blood vessels, C – cilia, D – desmosomes, GE – glandular epithelium, LE – luminal epithelial cell, MV – microvilli, MVBs – multivesicular bodies, N – nucleus, SE – secretome, TJ – tight junctions, UL – uterine lumen, Vs – vesicles

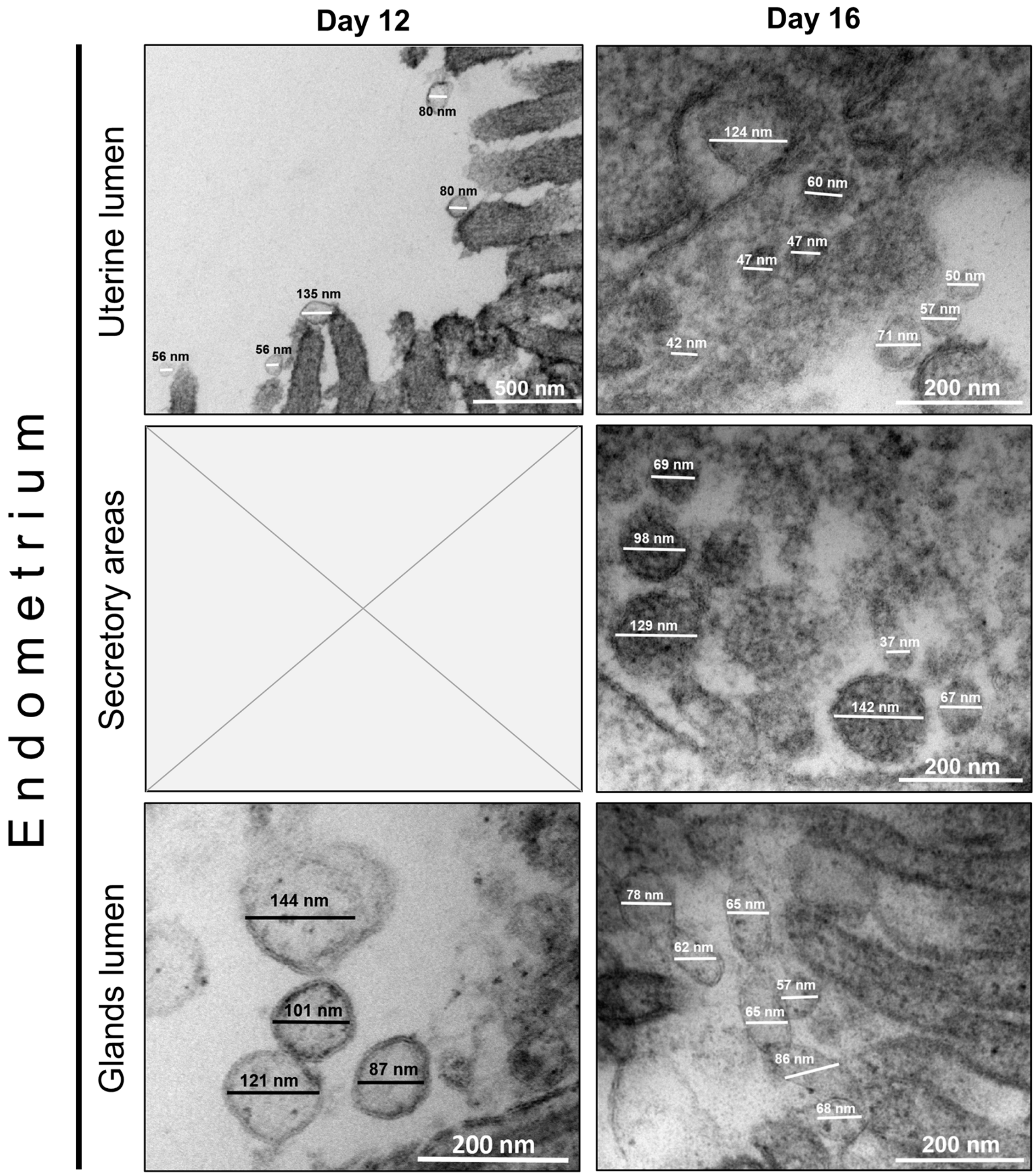

Supplementary Figure 4. Overview of micrographs showing heterogeneous population of extracellular vesicles found in endometrial compartments on day 12 and 16 of pregnancy. Apparent heterogeneity in size is visible, as indicated by diameter of vesicles enclosed by a lipid bilayer.

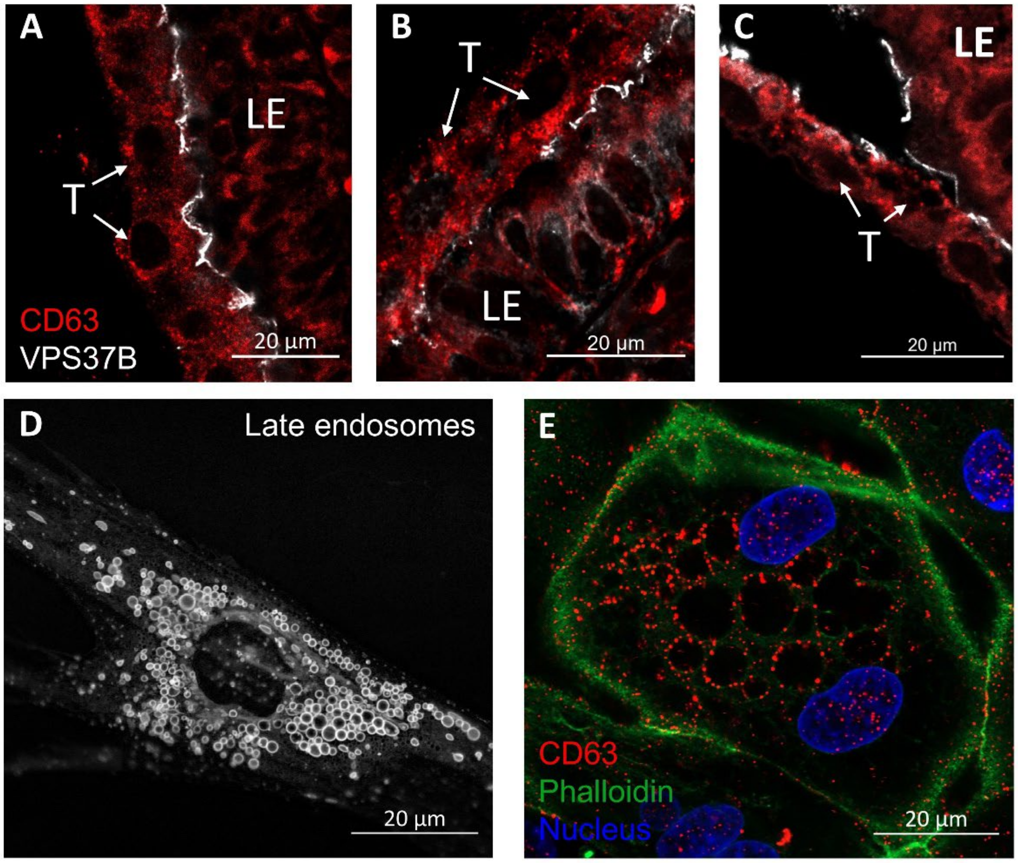

Supplementary Figure 5. CD63 appearance in the endocytic membrane transport pathway. **(A-C)** Distribution of CD63 (red) and VPS37B (white) at the embryo-maternal interface at day 16 of pregnancy. LE – luminal epithelium, T – trophectoderm. **(D)** Live-imaged primary luminal epithelial cells transfected with baculovirus. The BacMam-mediated fusion construct of Rab7 (a marker of late endosomes/MVBs) with emerald green fluorescent protein (EM-GFP) was used as a specific targeting tool for labeling late endosomes and MVBs (grey scale). **(E)** Colocalization of CD63 (red) and F-actin filaments visualized with phalloidin (green) in primary luminal epithelial cell. DAPI was used to stain nucleus.

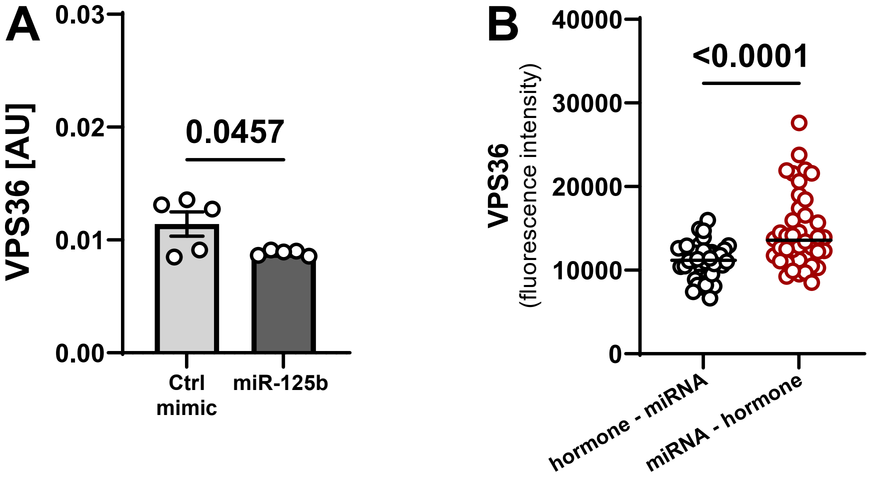

Supplementary Figure 6. miRNA effect on target gene and protein abundance. **(A)** miR-125b-5p affect the expression of target gene - VPS36 ($p = 0.0457$, unpaired T- test) in luminal epithelial cells. Data are presented as boxplots with means ± SEM. mRNA expression was normalized to HPRT1 (AU), identified as the best reference gene by NormFinder algorithm. **(B)** Fluorescence intensity of VPS36 signal in cell borders after miR-125b-5p delivery to luminal epithelial cells. Comparison between treatments revealed significant increase of VPS36+ puncta when embryonic signals were delivered after miRNA ($p < 0.0001$, unpaired T-test).

Supplementary Table 1. List of primary and secondary antibodies used in immunofluorescent staining of endometrial/trophoblast tissue and luminal epithelial cells.

	Primary antibodies	Host	Description	Dilution	Catalog number	Company
TISSUE ENDOMETRIUM/ TROPHOBLAST	Anti - VPS37B	Rabbit	Polyclonal	1:50	ab122450	Abcam
	Anti - Rab27A	Mouse	Monoclonal	1:50	ab55667	Abcam
	Anti - Alix	Rabbit	Polyclonal	1:50	PA5-52873	Invitrogen
LUMINAL EPITHELIAL CELLS	Anti - VPS36	Rabbit	Polyclonal	1:50	PA5-58905	Invitrogen
	Anti - CD63*	Mouse	Monoclonal	1:50	ab193349	Abcam
	Anti - Cytokeratin	Mouse	Monoclonal	1:50	C-9687	Sigma

*used also for TISSUE ENDOMETRIUM/TROPHOBLAST staining

Secondary antibodies	Host	Conjugated dye	Dilution	Catalog number	Company
Anti-Mouse IgG	Donkey	Cy3	1 : 3000	715-165-150	Jackson Immuno-Research
Anti - Rabbit IgG	Donkey	Cy3	1 : 3000	715-165-152	Jackson Immuno-Research
Anti - Rabbit IgG	Goat	Alexa Fluor 488	1 : 3000	A-21206	Invitrogen

Supplementary Table 2. List of mimics used in miRNA and hormone treatment experiment.

miRNA mimic	Assay name	Assay ID	Mature miRNA Sequence	Catalog number
**miR-125b-5p**	hsa-miR-125b-5p	MC10148	UCCCUGAGACCCUAACUUGUGA	4464067
**control mimic**	*mir* Vana™ miRNA Mimic, Negative Control #1			4464059

Supplementary Table 3. List of genes and TaqMan gene expression assays used in real-time PCR.

Gene symbol	Gene name	Group/family/complex name	Assay ID
Rab11A	Rab11a	RAS Oncogene Family	Ss03379468_u1
Rab11B	Rab11B	RAS Oncogene Family	Ss04322113_m1
Rab27A	Rab27A	RAS Oncogene Family	Ss03392039_m1
Rab7B	Rab7B	RAS Oncogene Family	Bt03251067_m1
Rab8B	Rab8B	RAS Oncogene Family	Bt03243268_m1
VPS22 (SNF8)	Vacuolar-Sorting Protein SNF8 homolog	ESCRT-II Complex Subunit	Bt03245176_m1
VPS25	Vacuolar-Sorting Protein 25 homolog	ESCRT-II Complex Subunit	Hs01003777_gH
VPS28	Vacuolar-Sorting Protein 28 Homolog	ESCRT-I Complex Subunit	Hs01598026_m1
VPS36	Vacuolar-Sorting Protein 36 homolog	ESCRT-II Complex Subunit	Bt03262017_m1
VPS37B	Vacuolar-Sorting Protein 37 homolog	ESCRT-I Complex Subunit	Ss04324043_m1
VPS4A	Vacuolar-Sorting Protein 4 homolog A	ESCRT-associated protein	Ss04324243_m1
ALIX (PDCD6IP)	Programmed Cell Death 6 Interacting Protein	ESCRT-associated protein	Hs00994345_m1
VAMP8	Vesicle Associated Membrane Protein 8	ESCRT-associated protein	Bt03225817_m1
ESR1	Estrogen Receptor 1	Nuclear Receptor Subfamily 3 Group A	Ss03383398_u1
ESR2	Estrogen Receptor 2	Nuclear Receptor Subfamily 3 Group A	Ss03391479_m1
ACTB	Beta-actin	Reference gene	Ss03376081_u1
GAPDH	Glyceraldehyde 3-phosphate Dehydrogenase	Reference gene	Ss03375435_u1
HPRT1	Hypoxanthine–guanine Phosphoribosyl transferase 1	Reference gene	Ss03388274_m1
